# Detection of Near-Threshold Sounds is Independent of EEG Phase in Common Frequency Bands

**DOI:** 10.3389/fpsyg.2013.00262

**Published:** 2013-05-14

**Authors:** Benedikt Zoefel, Peter Heil

**Affiliations:** ^1^Leibniz Institute for NeurobiologyMagdeburg, Germany; ^2^Center for Behavioral Brain SciencesMagdeburg, Germany

**Keywords:** EEG, oscillation, phase, entrainment, delta-band, alpha-band, auditory, detection threshold

## Abstract

Low-frequency oscillations in the electroencephalogram (EEG) are thought to reflect periodic excitability changes of large neural networks. Consistent with this notion, detection probability of near-threshold somatosensory, visual, and auditory targets has been reported to co-vary with the phase of oscillations in the EEG. In audition, entrainment of δ-oscillations to the periodic occurrence of sounds has been suggested to function as a mechanism of attentional selection. Here, we examine in humans whether the detection of brief near-threshold sounds in quiet depends on the phase of EEG oscillations. When stimuli were presented at irregular intervals, we did not find a systematic relationship between detection probability and phase. When stimuli were presented at regular intervals (2-s), reaction times were significantly shorter and we observed phase entrainment of EEG oscillations corresponding to the frequency of stimulus presentation (0.5 Hz), revealing an adjustment of the system to the regular stimulation. The amplitude of the entrained oscillation was higher for hits than for misses, suggesting a link between entrainment and stimulus detection. However, detection was independent of phase at frequencies ≥1 Hz. Furthermore, we show that when the data are analyzed using acausal, though common, algorithms, an apparent “entrainment” of the δ-phase to presented stimuli emerges and detection probability appears to depend on δ-phase, similar to reports in the literature. We show that these effects are artifacts from phase distortion at stimulus onset by contamination with the event-related potential, which differs markedly for hits and misses. This highlights the need to carefully deal with this common problem, since otherwise it might bias and mislead this exciting field of research.

## Introduction

Oscillatory ongoing neural activity on macro-, meso-, and microscopic scales appears to be highly relevant for information processing in various brain structures (Klimesch, 1999; Hutcheon and Yarom, 2000; Engel et al., 2001; Linkenkaer-Hansen et al., 2004; Becker et al., 2011; Cohen, 2011; Scheeringa et al., 2011; Zoefel et al., 2011). Both power and phase of neural oscillations in different frequency bands matter (e.g., Fries et al., 2001; Lakatos et al., 2005; Rajkai et al., 2008; Schyns et al., 2011; Sauseng, 2012) and are assumed to reflect the instantaneous state of neural excitability (Arieli et al., 1996; Supèr et al., 2003; Buzsáki and Draguhn, 2004; Fiser et al., 2004; Jensen et al., 2012). Moreover, in humans the probability of detecting near-threshold stimuli seems to depend on the phase of EEG oscillations. The detection of weak somatosensory stimuli was found to correlate with the phase of infra-slow (<0.1 Hz) EEG oscillations (Monto et al., 2008) and that of visual stimuli with the phase in the δ- (1–4 Hz) or α-band (7–12 Hz) at, and preceding, stimulus onset (Busch et al., 2009; Mathewson et al., 2009; Busch and VanRullen, 2010; Cravo et al., 2013). Similarly, the probability of eliciting phosphenes by transcranial magnetic stimulation of occipital cortex varied with α-phase (Dugué et al., 2011; Romei et al., 2012). The detection of near-threshold auditory targets by humans has been reported to depend on EEG δ-, θ- (4–7 Hz), and α-phase when the oscillations are driven by background sounds or oscillating transcranial direct current stimulation (o-tDCS). Neuling et al. (2012) reported variation of the probability of detecting an auditory target in noise with the phase of α-oscillations entrained by o-tDCS at 10 Hz. In contrast, Ng et al. (2012) found that detection of auditory targets in noise depended on power and phase in the δ/θ-, but not the α-band. Henry and Obleser (2012) had subjects detect gaps in 3-Hz frequency-modulated sounds and found performance to vary with neural δ-oscillations entrained by the stimulus. Entrainment of δ-oscillations to audible and attended sounds, presented at regular or slightly jittered intervals, had been reported previously in monkeys and humans (Lakatos et al., 2005, 2008; Schroeder et al., 2008; Besle et al., 2011; Gomez-Ramirez et al., 2011). Furthermore, phase entrainment of δ-oscillations was associated with decreased reaction times (Stefanics et al., 2010). These findings, together with the fact that the δ-band covers important temporal scales of human speech (Giraud and Poeppel, 2012; Peelle and Davis, 2012), suggest an important role of the δ-phase, but possibly also of θ- and α-phases (see also Thorne et al., 2011), for auditory perception.

Here, we investigate whether detection of near-threshold auditory stimuli in quiet, whose underlying mechanisms are still debated (see, e.g., Viemeister and Wakefield, 1991; Eddins and Green, 1995; Heil and Neubauer, 2003; Verhey, 2010; Heil et al., 2011, 2013a,b; Meddis and Lecluyse, 2011; Pohl et al., 2013), depends on the phase of EEG oscillations in humans. Based on the aforementioned findings, we expected detection to vary with the phase of EEG oscillations, particularly in the δ-, θ-, and α-bands. Yet, in our paradigms, employing both irregular and regular, and hence predictable, stimulus times, detection probability was independent of phase at frequencies ≥1 Hz, irrespectively of stimulus predictability. In the regular condition, the EEG signal oscillated with the frequency corresponding to the rate of stimulus presentation, reflecting phase entrainment. The amplitude of this oscillation was higher, though not significantly, prior to detected than to undetected stimuli, indicating a relationship between the entrained oscillation and stimulus detection. Phase entrainment was paralleled by significantly decreased reaction times. Notably, application of acausal, but common, analysis algorithms produced artificial phase entrainment and dependences of detection probability on EEG δ-phase.

## Materials and Methods

### Participants

Forty-one subjects, including the two authors, took part in the study. Twenty-two (11 females, age range 20–56 years, median 24 years) participated in the first experiment and twenty-three (13 females, age range 22–45 years, median 26 years) in the second. Four subjects participated in both experiments. The study was approved by the Ethics Committee of the Otto-von-Guericke University Magdeburg. Subjects gave written informed consent and all but one (the second author) received monetary reward for participation. None of them reported neurological or hearing disorders. Data of four subjects (one male, three females; two from each experiment) were excluded from further analyses, in one case due to an excessive probability of false alarms (10.6%), far beyond the 0.75 quantile of the distribution (0.8%), in the other three cases due to excessive eye-movement artifacts.

### Experimental procedures

#### Overview

During the experiments, the subjects sat comfortably in an electrically shielded and sound attenuated double-walled spacious booth (Industrial Acoustic Chambers, Niederkrüchten, Germany), while their EEG was recorded. Brief and soft auditory stimuli were presented at irregular inter-stimulus intervals (ISIs) (Experiment 1), or at irregular and regular ISIs (Experiment 2), and subjects were instructed to press a button every time they detected a stimulus. The experiments were partitioned into blocks of 10 min each, with breaks in between, and for each experiment subjects completed 6–8 blocks, depending on their motivation. In Experiment 2, blocks of successive stimuli with irregular ISIs (“irregular condition”) alternated with blocks of successive stimuli with regular ISIs (“regular condition”) (see below). Other than that, Experiments 1 and 2 were essentially identical.

#### EEG recordings

The EEG was recorded with 32 Ag/AgCl electrodes, placed in an elastic cap (Easycap, Falk Minow Services, Munich, Germany) according to the international 10–20 system. The signals were amplified by a 32-channel amplifier system (BrainAmp, Brain Products GmbH, Munich, Germany), analog filtered between 0.01 and 250 Hz, and digitally sampled at a rate of 500 Hz. The reference and ground electrodes were located on the nose and at FCz, respectively, and the electrooculogram (EOG) was recorded by two electrodes, one below and one on the outer canthus of the right eye. Electrode impedances were kept below 10 kΩ.

#### Auditory stimuli

The auditory stimuli were pure tones with a carrier frequency of 3.125 kHz and a total duration of 12.48 ms (i.e., 39 carrier periods), including Hanning onset and offset ramps of 4.16 ms (i.e., 13 carrier periods) each. The stimuli were loaded into the buffer of a data acquisition board (PCI-6221, National Instruments) and triggered by the digital output port of a BNC block (BNC-2120, National Instruments). They were presented diotically via Sennheiser HDA 200 headphones, at near-threshold levels. The precise sound pressure levels (SPLs) used for each subject depended on the subject’s detection threshold for this stimulus, which was determined (in dB SPL; i.e., dB re 20 μPa) just prior to the start of, and in the same setting as, the main experiment. For this purpose, we used the method of limits (Gescheider, 1997). Here, the tone was presented diotically at intervals of 2 s. From one presentation to the next, the tone’s SPL either decreased from a clearly audible SPL or increased from an inaudible level, in steps of 1 dB. The subjects were instructed to press a button when they could no longer hear the tone (for the decreasing level sequences) and when they started to hear it again (for the increasing level sequences). For each subject, five decreasing and five increasing level sequences were used in alternation. The mean SPL during the 10 button presses was defined as the threshold SPL for this subject. In the immediately following main experiment, the tones were presented at this threshold SPL (referred to as 0 dB) and at 2 dB below (−2 dB) and 2 dB above (+2 dB) this value, with approximately equal probabilities and in random order. For some subjects, it was necessary to slightly alter the SPLs during the experiment (by ±1–2 dB), since otherwise they would have ended up detecting fewer than 25% or more than 75% of the tones presented. In these subjects, subtle shifts in sensitivity during the time between threshold determination and main experiment might have occurred. Shifts in sensitivity can occur over various time scales and can be quite substantial (Heil et al., 2006).

#### Timing of auditory stimuli: experiment 1

Phase entrainment of δ-oscillations to discrete auditory stimuli can be observed when they are presented as a train and the ISI is constant (767 ms, Lakatos et al., 2005; Schroeder et al., 2008; 1500 ms, Gomez-Ramirez et al., 2011). Phase entrainment is visible even with a moderately jittered ISI (650 ± 150 ms; Lakatos et al., 2008; 667 ± 150 ms, Besle et al., 2011), although the entrainment is less pronounced than with a constant ISI. If stimuli were presented at regular ISIs and phase entrainment occurred, the vast majority of stimuli would coincide with one particular phase and very few, if any, with the remaining phases. For determining whether detection depends on phase, a regular ISI design thus appears sub-optimal. Of course, stimuli could be presented at irregular ISIs but the degree of “randomness” sufficient to prevent phase entrainment is unknown. Therefore, to circumvent this problem in our first experiment, we aimed at presenting the stimuli at four different phases, covering the full cycle and thus being separated by π/2 (or 90°). We chose ±π, π/2, −π/2, and 0, defined with respect to a cosine wave. Note that 0 = 2π, π=-π, $\frac{\pi }{2}=-3\frac{\pi }{2}$, $-\frac{\pi }{2}=3\frac{\pi }{2}$. We focused on a frequency in the δ-band, specifically 2 Hz. In order to achieve this goal, 2 Hz phases were estimated online during the actual recording, at electrode Cz. The approach of estimating phase online has been used before (e.g., Dustman and Beck, 1965; Varela et al., 1981; Rice and Hagstrom, 1989; Kruglikov and Schiff, 2003). Most of these studies relied on trigger systems for stimulation, automatically presenting stimuli at the trough or peak of EEG waves or with certain delays to target different phases. In our study, a sliding Fast Fourier Transform (FFT) algorithm, updated every 20 ms, was used to estimate phase online. For the FFT, the duration of the time window was 1.024 s (512 sample points, including Hanning window to avoid edge effects). Computational and conductional delays resulted in a final delay of 1.1 s, equal to 1.2 times the period (or $2\pi +\frac{2\pi }{5}$) of a 2 Hz oscillation. In order to present a tone, or a catch, at the desired phases of the 2 Hz oscillation, the delay corresponding to the 2π/5 fraction of the 2 Hz period needed to be compensated for. Consequently, whenever the software detected the phases $\pm \pi -\frac{2\pi }{5}$, $\frac{\pi }{2}-\frac{2\pi }{5}$, $-\frac{\pi }{2}-\frac{2\pi }{5}$, and $0-\frac{2\pi }{5}$, a tone or a catch was presented so that it would coincide with the desired phases of ±π, π/2, −π/2, and 0.

**Figure 1:**
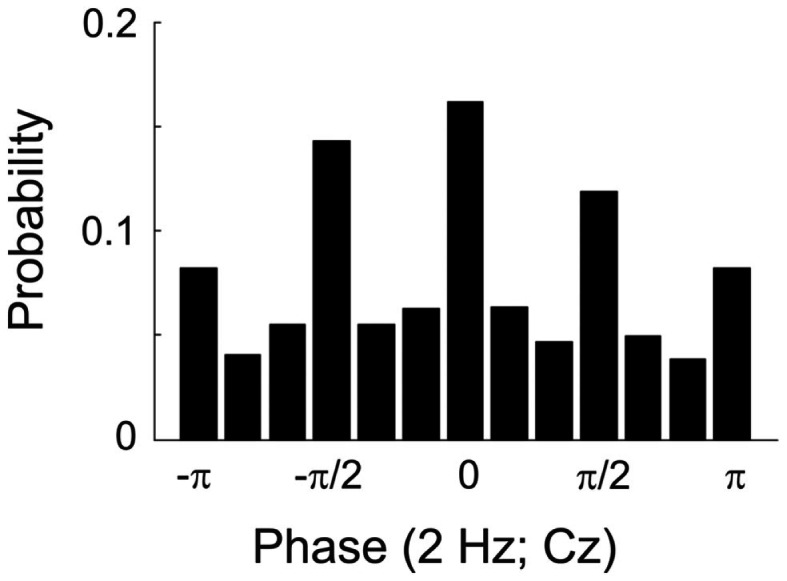
**Histogram showing the probability of presenting a tone at particular phases of a 2-Hz oscillation in the EEG (at electrode Cz) in Experiment 1**. The full cycle is divided into 12 bins, π/6 or 30° wide. Bins are centered at the values specified. Note that the 12th bin includes both π and −π and is therefore depicted twice.

The degree to which this procedure was successful can be appreciated from Figure 1, which plots the probability of tone presentation as a function of the phase at 2 Hz, grouped into 12 bins of π/6 (or 30°) each. The phase at the time of tone presentation was derived offline from the EEG signal at electrode Cz by means of a FFT (including Hanning window) in a 1.024-s pre-stimulus window ending at stimulus onset. Figure 1 reveals that 50.6% of the tone presentations fell into one of the four desired phase bins, well above the chance level of 33.3%. We assume that the unavoidable delays between phase estimation online and tone presentation prevented an even better match of the achieved with the desired phases.

**Figure 2:**
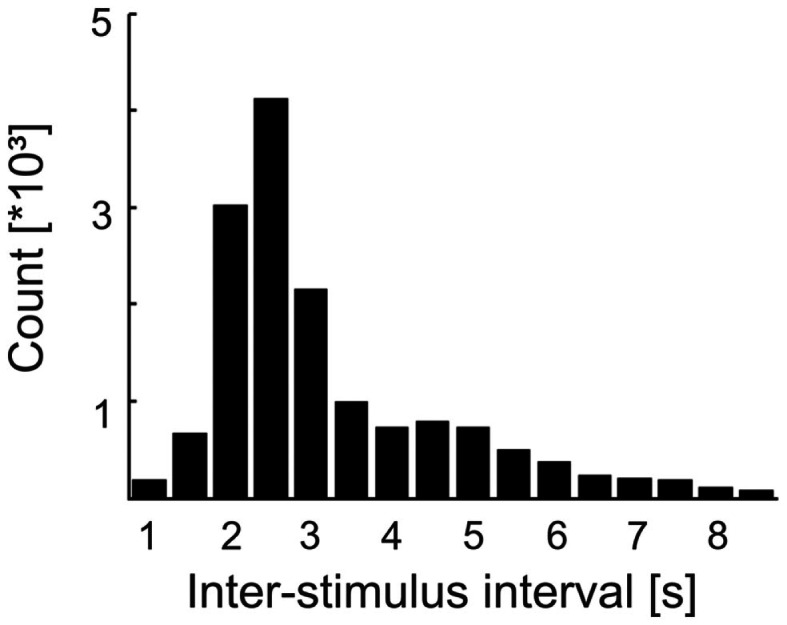
**Distribution of inter-stimulus intervals (ISIs) in Experiment 1**. Bin width is 0.5 s and bins are centered at the values specified. Note the skew and width of the distribution. Combined with the fact that about 50% of the tones were missed by the subjects, the times of tone occurrence were unpredictable.

Following each tone or catch, the software restarted phase estimation with a random delay between 1 and 1.5 s. Also, some additional and variable time elapsed from the restart of the software to the first detection of one of the four desired phases. In addition, the presentation of the next stimulus could be delayed by the frequent (25% of the cases), but random, insertion of catch trials. Consequently, the ISIs (defined here as the intervals between the onsets of successive tones) were rather long (mean: 3.38 s; median: 2.66 s) and their distribution was broad (SD: 1.88 s; interquartile range: 2.21–4.03 s), i.e., ISIs were highly variable (Figure 2). Furthermore, about 50% of the tones presented were missed by the subjects (see Results). Therefore, the times of occurrence of the tones were unpredictable for the subjects.

#### Timing of auditory stimuli: experiment 2

In Experiment 2, we presented stimuli at irregular ISIs, as in Experiment 1, as well as at regular ISIs. Stimuli at irregular (“irregular condition”) and regular ISIs (“regular condition”) were presented in alternating blocks. The choice of the first block across subjects was by chance. In the irregular condition, ISIs were drawn randomly from a distribution identical to that obtained in Experiment 1 (Figure 2). Consequently, the irregular condition of Experiment 2 was very similar to Experiment 1. In the regular condition, stimuli were presented at a constant ISI of 2 s.

### Data analyses

All statistical analyses were performed in MATLAB. The Circular Statistics Toolbox for MATLAB (Berens, 2009) was used for circular statistics (Rayleigh’s Test, Kuiper’s Test, Watson–William-Test).

#### Analysis of behavioral data

Based on previous experience with similar response tasks (Heil et al., 2006; Tiefenau et al., 2006), a tone was considered as detected, and the response classified as a hit, if the subject pressed the response button within 1.2 s following tone onset. If the reaction time was longer, or the button was not pressed at all before the onset of the next tone, the response or lack thereof was classified as a miss. In Experiment 1, a button press within the corresponding 1.2 s time window of a catch trial was defined as a false alarm and the lack of a button press in that interval as a correct rejection. Reaction times were measured with an accuracy of 2 ms.

#### Calculation of event-related potentials

Event-related potentials were calculated separately for detected (hits) and undetected tones (misses) as well as for catch trials (in Experiment 1). The epochs selected for computing the ERP covered the interval from −500 ms to +800 ms relative to tone onset in Experiment 1 and from −1000 ms to +800 ms in Experiment 2, to more clearly document the slow negative potentials shifts preceding stimulus onsets in the regular condition (see Results). ERP epochs were baseline corrected, by subtracting the mean value of the first 200 ms of the respective pre-stimulus window, before averaging them in order to reveal grand-mean ERPs. Epochs were first averaged across trials and then across subjects to compensate for different numbers of hits and misses in different subjects. For display purposes only, ERPs were smoothed by filtering (second-order lowpass Butterworth filter, cutoff 20 Hz).

#### Offline estimation of phase at tone onset

In a first step, we used two causal algorithms in order to estimate the phase at tone onset: (1) causal bandpass (BP) filtering with subsequent Hilbert Transformation (HTF) and (2) FFT. These algorithms rely exclusively on pre-stimulus data to estimate the phase at tone onset. For both algorithms, data epochs (see below) were automatically and visually screened for eye blinks and other movement artifacts and contaminated epochs were discarded. The remaining epochs were baseline corrected, by subtracting the mean value of a 1-s pre-stimulus window, before being processed further. For algorithm (1), BP filtering (second-order Butterworth filter; MATLAB function *filter*) was applied to a 2-s window, centered at stimulus onset. Because the response of a causal filter depends only on data lying in the “present” or the “past,” the phase at stimulus onset is estimated by pre-stimulus data only. Before BP filtering, a Hanning window was applied to the 2-s window in order to avoid edge effects. Different passbands of the filter were used to explore possible effects of phase in different EEG bands (for example, passbands of 1–4 Hz for δ and of 7–12 Hz for α). For algorithm (2), FFT was performed on the signal in a 1.024-s window (512 sample points) ending at tone onset, after application of a Hanning window to avoid edge effects.

Both algorithms include convolution with an impulse response (shaped as a windowed sinusoid in the case of FFT). Still, the frequency range which affects the estimated phase is typically much smaller for FFT [and wavelet transformation (WTF), see below] than for BP filtering with HTF, as BP filters become extremely long for narrow passbands. In our case, the frequency resolution of the FFT was 1 Hz, whereas the widths of the filter passbands were ≥4 Hz. Thus, the two algorithms cannot be expected to yield identical results.

In many studies, where the phase of an oscillation at the time of stimulus onset has been of interest, a time window of, e.g., 2–4 s has been centered on each stimulus onset. The phase at stimulus onset was then estimated from these epochs by acausal BP filtering and HTF or by a WTF (e.g., Will and Berg, 2007; Monto et al., 2008; Busch et al., 2009; Reimer and Hatsopoulos, 2010; Saleh et al., 2010; Stefanics et al., 2010; Besle et al., 2011; Gomez-Ramirez et al., 2011; Henry and Obleser, 2012; Ng et al., 2012; Cravo et al., 2013). We therefore decided to also apply similar algorithms to estimate phase at stimulus onset. For illustrations, we selected a 2-s time window, and in one instance also a 1-s window, centered on each tone onset. Again, epochs containing artifacts were removed and the remaining epochs were baseline corrected. In order to estimate phase at tone onset, we applied (1) zero-phase, i.e., acausal, BP filtering (second-order Butterworth filter, passbands as above; MATLAB function *filtfilt*; including Hanning window) with subsequent HTF and (2) WTF (complex Gaussian wavelets with frequencies from 1 to 125 Hz, in steps of 1 Hz).

Note that the BP filters used for causal and acausal filtering are identical but they process the data in different ways. The output of the causal filter only depends on “current” or “past” inputs, whereas that of the acausal filter also depends on data lying in the “future”.

#### Analysis of detection probabilities as a function of phase

To display the probability of tone detection as a function of phase, the 360° cycle was divided into 12 bins of 30° each. The detection probability in a given bin was calculated as the number of tones detected divided by the number of tones presented in that bin, thus compensating for unequal numbers of tones presented in different phase bins. Detection probabilities were determined individually for each subject. These probabilities were then averaged across subjects in each phase bin.

If there were an effect of EEG phase on tone detection, the detection probability can be expected to be maximal if the tone occurred during a particular phase (“preferred” phase), and minimal if it occurred during another (“worst” phase), along with phases where the detection probability is above and below average, respectively. To examine whether detection probability was uniform or not, we applied Rayleigh’s Test for uniformity of phase data (Fisher, 1993). The vector strength, *r*, was calculated as

(1) $$r=\frac{1}{n}\sqrt{{\left(\overset{n}{\underset{i=1}{\sum }}\cos\;\alpha_{\text{i}}\right)}^{2}+{\left(\overset{n}{\underset{i=1}{\sum }}\sin\;\alpha_{i}\right)}^{2}}$$

Here, α is the phase angle and *n* is the number of observations. We used for *n* the average number of detected tones across subjects. This number was 415 for Experiment 1, 355 for the regular condition of Experiment 2, and 230 for the irregular condition of Experiment 2. The test statistic *z* is given by *z* = *nr*^2^. A significant test value indicates a non-uniform distribution of detection probabilities, with a “preferred” phase for tone detection.

We performed these analyses for the following frequency bands: δ (1–4 Hz), θ (4–7 Hz), α (7–12 Hz), lower α (8–10 Hz), upper α (10–12 Hz), lower β (13–18 Hz), upper β (19–30 Hz), and lower γ (25–40 Hz). Note that there is some heterogeneity in the literature regarding the frequency limits used to define different bands.

#### Analysis of the influence of power on detection probabilities as a function of phase

Effects of power in the α-band on detection of visual targets (Mathewson et al., 2009; Jensen et al., 2012) and in the δ/θ-band on detection of auditory targets (Ng et al., 2012) have been demonstrated. Therefore, we analyzed power in the frequency bands from δ to lower γ (as defined above). Power was extracted from single trials by means of FFT in the pre-stimulus window of 1.024 s (*N* = 512, including Hanning window). We divided the trials, for each band separately, into high- and low-power trials, using a median split. The distributions of detection probability as a function of phase [obtained from the same analysis; see algorithm (2) above] for high- and low-power trials of the respective frequency bands were compared using Kuiper’s Test, the circular analog of the Kolmogorov–Smirnov-Test.

#### Analysis of phase entrainment

If there were an entrainment of phase to the times of stimulus presentation, the distribution of phase at stimulus onset would be non-uniform, with a peak at the “entrained” (“preferred”) phase. To examine this issue, we therefore determined the distribution of phase at tone onset, individually for each subject. Phase was divided into 12 bins. The individual phase distributions were first normalized in order to correct for unequal numbers of stimuli and then averaged across subjects, separately for the random and the regular condition and for hits and misses. We then applied Rayleigh’s Test, with the average numbers of hits and misses across subjects being *n*_hits_ = 355 and *n*_misses_ = 303 in the regular condition and *n*_hits_ = 230 and *n*_misses_ = 229 in the irregular condition. A significant test value would indicate a non-uniform distribution of phase at stimulus onset, i.e., the existence of a “preferred” phase, and therefore, by definition, of phase entrainment.

#### Analysis of the influence of phase on reaction times

In order to investigate the possibility that the phase of an EEG oscillation at the time of stimulus presentation might have an effect on reaction time (Stefanics et al., 2010), the hit trials of all subjects were pooled and then split according to the median of the reaction times, as suggested by VanRullen et al. (2011). The mean phases (estimated using causal BP filtering and HTF) in the frequency bands from δ to lower γ of the two subgroups of hit trials were then calculated and compared using the Watson–William-Test (tests equal means of circular data).

## Results

### Experiment 1

#### Behavioral results

The mean of the thresholds for the brief tone stimulus across the 20 subjects was 7.6 dB SPL. During the actual experiment, these tones were presented to each subject at three different near-threshold SPLs separated by 2 dB. The mean detection probability, computed by averaging the individual detection probabilities to correct for unequal numbers of tones presented to each subject, was 49.8 ± 6.0%. As expected, the mean detection probability increased with increasing sound level, from 22.4 ± 6.6% via 50.4 ± 9.2 to 77.3 ± 9.6%. A fit of these data with a cumulative normal distribution as a model for the psychometric function yielded a mean of 7.9 dB SPL and a SD of 2.9 dB. The SD, which reflects the steepness of the psychometric function, is very similar to those obtained with much more comprehensive data in a previous study (Heil et al., 2006).

The probability of false alarms was estimated by means of catch trials. The median probability across the 20 subjects was 0.56% with an interquartile range from 0.19 to 0.80%. Due to these low probabilities, we refrained from correcting the reaction time distributions for false alarms, unlike in previous studies (Heil et al., 2006; Tiefenau et al., 2006).

**Figure 3:**
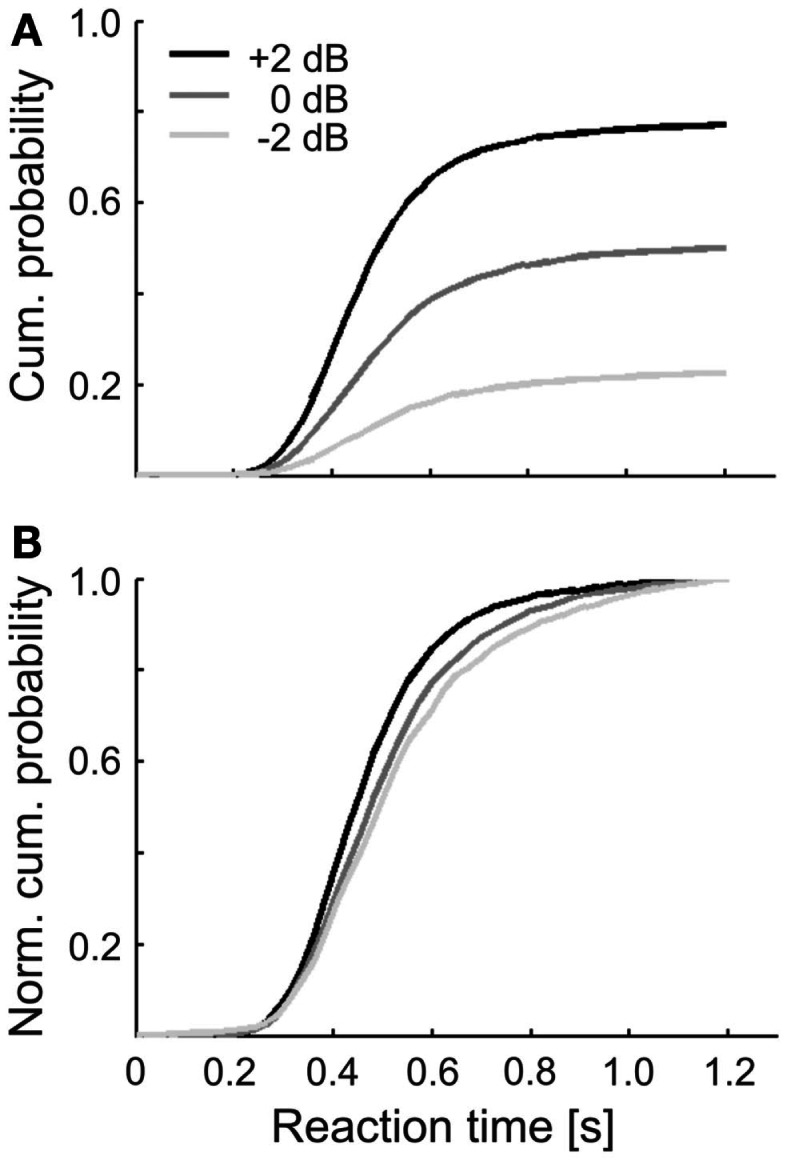
**(A)** Cumulative distributions of simple reaction times in response to the tones of the three sound levels in Experiment 1. Reaction times were accumulated across all hit trials and subjects. **(B)** Same distributions as in **(A)**, but normalized to 1 to illustrate the sound level related differences in their shapes. Legend in **(A)** applies to both panels.

Figure 3A shows the cumulative probabilities of the reaction times, separately for the three sound levels. The three distributions reach different asymptotic levels, due to the increase in detection probability with increasing sound level. In addition, their shapes differ somewhat. This is emphasized in Figure 3B, where the same distributions are shown after normalizing them to 1. The median of these normalized distributions decreased with increasing sound level, as expected (see Heil et al., 2006, and references therein). The decrease, from 0.50 s at −2 dB via 0.49 s at 0 dB to 0.45 s at +2 dB, is larger than the tone duration and thus larger than can be accounted for by purely sensory components (Heil et al., 2006). The median of all reaction times pooled across subjects and sound levels was 0.47 s with an interquartile range from 0.39 to 0.58 s.

#### The ERPs for hits and misses differ

**Figure 4:**
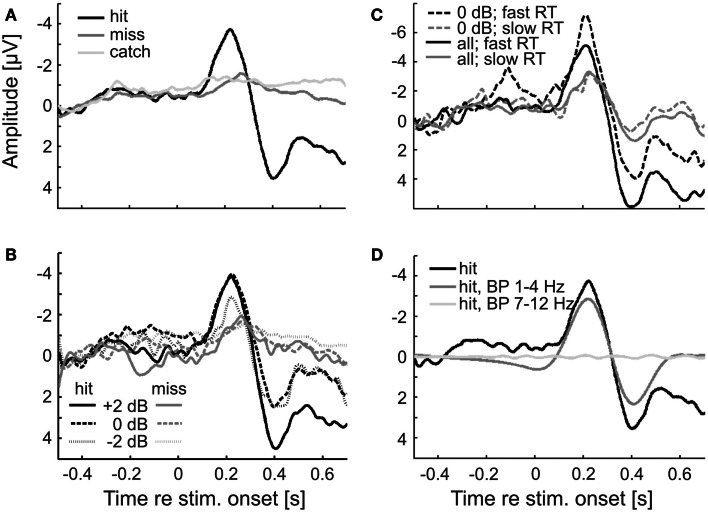
**ERPs for hits and misses differ**. **(A)** ERPs (at electrode Cz) for hits and misses averaged across all subjects, trials, and sound levels in Experiment 1. ERPs from catch trials are also shown. **(B)** ERPs to hits and misses separately for the three sound levels. **(C)** ERPs separated according to trials with fast and slow reaction times (median split), averaged across all sound levels (continuous lines), or just across one (0 dB; dashed lines). **(D)** BP filtering of the single-trial ERPs to hits with a zero-phase filter results in phase distortion at stimulus onset when the passband of the filter matches the δ-band but not when it matches the α-band. The black curve shows the average ERP to hits, without zero-phase BP filtering [reproduced from **(A)**]. The dark and light gray curves show the ERPs after zero-phase BP filtering of single trials with passbands of 1–4 Hz and 7–12 Hz, respectively.

Figure 4 shows the ERPs for hits and misses, from the EEG signals at electrode Cz. The ERPs shown in Figure 4A were derived by averaging the signals across all hit trials (black) and all miss trials (dark gray), irrespective of sound level. These ERPs clearly differ. The ERP for hits contains both a prominent negative and a prominent positive component, peaking at about −4 μV and 200 ms and 4 μV and 400 ms post-stimulus onset, respectively. In contrast, the ERP for misses is rather flat. Only a modest negative peak of −1 μV at about 250 ms can be discerned, but no positive component. We presume that the early negative peak to hits and misses is the common N100, delayed due to the low sound levels, and the later positive peak, present to hits but absent to misses, the delayed P300. We cannot entirely exclude the possibility that this late positive peak also contains (preparatory) motor response components. However, its strong dependence on sound level (see Figure 4B and text below) argues against it being a pure motor response. The average potential obtained from catch trials hardly deviates from zero, as expected (Figure 4A; light gray). Because of the low probability of false alarms, we refrained from a separate analysis of that potential for false alarms and correct rejections. Note that during the pre-stimulus (and pre-catch) periods, the average EEG signals of hit and miss trials (and of catch trials) are very similar. This finding already points to a lack of a systematic relationship between the pre-stimulus EEG and stimulus detection. If there were such a relationship, the pre-stimulus EEG signals of hit and miss trials might be expected to differ.

Figure 4B shows the ERPs for hits and misses separately for the three sound levels. For hits, the absolute amplitudes of both the early negative component and the later positive component increase with increasing sound level. For misses, the absolute amplitude of the early negative component also increases with increasing sound level, but only slightly. Comparisons of the ERPs for hits and misses evoked by identical tones and sound levels reveal that absolute peak amplitudes are higher and peak latencies shorter for hits than for misses. The absolute peak amplitude of the early negative ERP component evoked by tones of the lowest sound level when they were detected is even higher than that evoked by tones of the highest sound level when they were not detected.

In order to examine whether there is a relationship between the ERP and reaction time, we averaged the EEG signals separately for trials with fast and with slow reaction times (below and above the median reaction time, respectively). The results are shown in Figure 4C. Interestingly, both the early negative and the late positive components of the ERP are much larger on trials with fast compared to trials with slow reaction times. To explore the possibility that this result may have been due to an uneven distribution of sound levels across the two groups of trials, we performed the same analysis restricted to trials of a given sound level (0 dB, as defined in Materials and Methods). The result was the same: both ERP components are much larger on trials with fast reaction times. Our finding differs from results reported by Wilkinson and Morlock (1967) who found no correlation between reaction time and ERP components evoked by acoustic clicks.

#### EEG phase and tone detection

We examined whether the probability of detecting near-threshold tones depends on the estimated phase of EEG oscillations at tone onset, in all frequency bands from δ to lower γ, but for conciseness focus on our findings for the δ- and α-bands only. For the same reason, we report and illustrate only the results from electrode Cz, and note that those from other electrodes were similar. Also, we combined the results obtained with different sound levels, and note that the results were similar at each sound level when considered separately (apart from the differences in the mean detection probability described above).

##### EEG δ-phase and tone detection: causal algorithms

**Figure 5:**
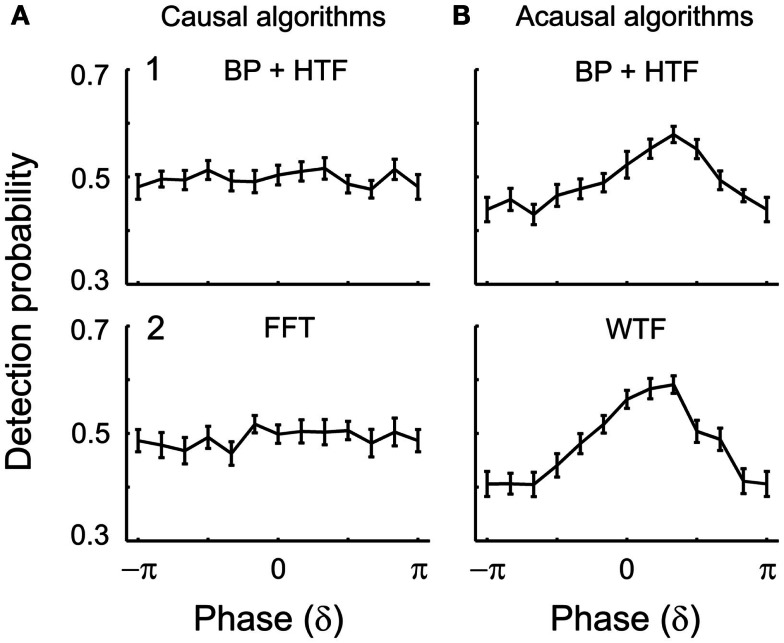
**Detection probability as a function of estimated δ-phase at the time of tone onset in Experiment 1**. The functions represent the mean detection probability across subjects and the error bars the SEM. In **(A)**, causal algorithms, and in **(B)**, acausal algorithms, were used with a 2-s window centered at tone onset. The algorithms used to estimate phase were (1) BP filtering (1–4 Hz) followed by HTF and (2) FFT or WTF (both shown for the representative frequency of 2 Hz). An apparent dependence of detection probability on phase emerges with the acausal algorithms which make use of post-stimulus data including the ERP (cf. Figure 4). In contrast, when phase is estimated by causal algorithms, detection probability is independent of δ-phase.

Figure 5 illustrates the results for the δ-band. The two panels (1,2) of Figure 5A show the mean detection probability as a function of δ-phase at tone onset. Phases were estimated by (1) causal BP filtering with subsequent HTF or (2) FFT (See Materials and Methods). For both algorithms, the distribution of the mean detection probability across phase does not differ from uniformity [BP/HTF: *z*(415) = 0.08, *p* = 0.93; FFT: *z*(415) = 0.51, *p* = 0.60]. For each phase bin, detection probability is about 50%. Thus, the mean detection probability of near-threshold tones in this experiment is independent of the EEG δ-phase at tone onset.

**Figure 6:**
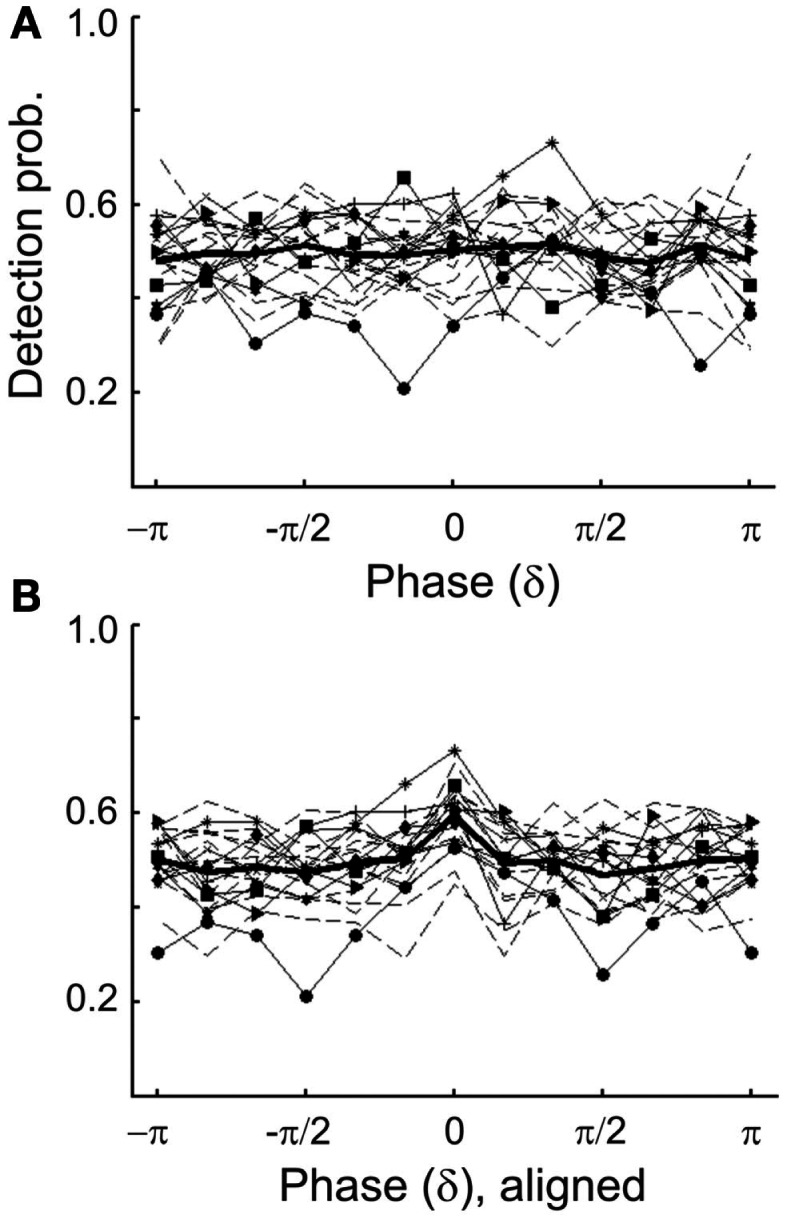
**(A)** Detection probability of individual subjects as a function of the estimated δ-phase at tone onset (thin lines) in Experiment 1. Phase was estimated by causal BP filtering (1–4 Hz) followed by HTF. The mean function (thick line) is the same as that in Figure 5A1. Different symbols connected by continuous lines identify those eight subjects for whom the distribution of detection probability deviated significantly from uniformity (*p* < 0.05). Note their erratic nature. Data from the remaining subjects are shown by broken lines. **(B)** Same individual functions as in **(A)**, but aligned such that the maximum detection probability of each subject coincides with the center bin. Note that, of course, the mean function now exhibits a peak at the center bin but is flat otherwise.

Because the detection probabilities shown in Figure 5A represent the means across subjects, it is conceivable that a significant dependence of detection probability on δ-phase might exist at an individual level. If the “preferred” phases of individual subjects differed widely, the observed lack of a significant dependence of the mean detection probability on δ-phase could have resulted from averaging out significant dependencies at the individual level. To examine this possibility, we explored the dependence of detection probability on phase also at the individual level. We only show the data with phase estimated by causal BP filtering and HTF, and note that those with phase estimated by FFT were very similar. Figure 6A plots the individual results (thin lines) and their average (thick line; same as in Figure 5A1). For 8 of the 20 subjects, the distributions of detection probability across phase differed significantly from uniformity (*p* < 0.05). However, the fluctuations of detection probability with phase appeared rather erratic, even in these eight subjects (functions with symbols in Figure 6A), without a common pattern across subjects. Nevertheless, we defined a “preferred” phase for each subject as that one which coincided with the highest detection probability. In Figure 6B, the individual functions are re-plotted, but are now shifted parallel to the phase axis, such that the individual “preferred” phases fall into the same bin (centered at 0), just as done in other studies (Busch et al., 2009; Ng et al., 2012). Now, of course, the average detection probability shows a peak in that bin. Notably, however, there is no average “worst” phase at or near the phase opposite the peak. In fact, apart from the peak, the function is rather flat, and Rayleigh’s Test does not allow rejecting the null hypothesis of a uniform phase distribution [*z*(415) = 1.07, *p* = 0.39].

Taken together, the results obtained with the causal algorithms suggest that under our experimental conditions tone detection is independent of the phase of EEG oscillations in the δ-band.

##### EEG δ-phase and tone detection: acausal algorithms

**Figure 7:**
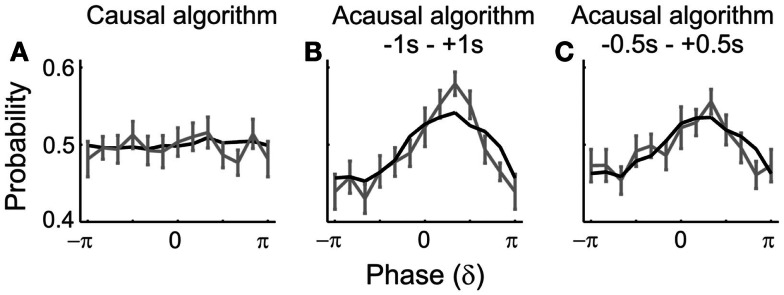
**Simulations confirm an artificial dependence of detection probability on δ-phase**. The black lines represent the “detection probabilities” derived from simulated data in which the “detection probability” is 50%, independent of the phase. For details of the simulation see Results. The gray lines and error bars represent the mean and SEM of the detection probabilities derived from the real data [those in **(A,B)** are the same as in Figures 5A1,B1; those in **(C)** are those derived with a time window of 1 s, centered on tone onset]. Phases at “tone” onset were extracted by causal **(A)** or acausal **(B,C)** BP filtering and HTF. Note the striking similarity of the phase-dependencies of detection probabilities derived from the real and the simulated data.

The two panels (1,2) of Figure 5B show the mean detection probability as a function of δ-phase at tone onset when phase was estimated by acausal algorithms. Phase was estimated by (1) zero-phase BP filtering with subsequent HTF and (2) WTF (See Materials and Methods). As mentioned above, such algorithms have been used in several other studies. Figure 5B shows the data obtained from a 2-s window centered at tone onset, but similar results were obtained with other window durations (for example, of 1 s; see Figure 7). For both algorithms, the distribution of mean detection probability across phase differs significantly from uniformity [zero-phase BP/HTF: *z*(415) = 9.37, *p* < 0.001; WTF: *z*(415) = 22.37, *p* < 0.001]. Detection appears to be best at a “preferred” phase. Detection probability decreases more or less continuously away from the “preferred” phase and reaches a minimum (“worst” phase) at or near the opposite phase. The resulting sinusoidal dependences of detection probability on the phase of an EEG oscillation, as well as the magnitudes of the effects, with detection probability varying from about 40% at the “worst” phase to 60% at the “preferred” phase, are similar to those reported in several other studies (Monto et al., 2008; Busch et al., 2009; Mathewson et al., 2009; Busch and VanRullen, 2010; Henry and Obleser, 2012; Ng et al., 2012; Cravo et al., 2013). The magnitude of the effect is equivalent to that expected for a change in sound level by about ±1 dB (i.e., in sound amplitude by about ±12%), as can be derived from the psychometric function.

However, the 2-s peri-stimulus time window used here comprises the ERP or substantial portions of it, as is readily seen from Figure 4. This also applies to other peri-stimulus time windows for which detection probability appeared to vary with δ-phase, such as the 1-s window. In such instances, it cannot be excluded that the ERP is “smeared” back in time (Sauseng et al., 2007; VanRullen, 2011; Rousselet, 2012; Widmann and Schröger, 2012) and so affects the phase estimates at the time of stimulus onset. This “smearing” of the ERP is shown in Figure 4D. Here, the signals from all individual hit trials were subjected to zero-phase filtering in the δ-band (same filter as for the extraction of phase) prior to averaging them. The shape of the average filtered ERP differs from that computed by averaging the unfiltered signals of all individual hit trials (black; same function as in Figure 4A) at the time of, and even long before, stimulus onset.

##### Apparent dependence of detection on δ-phase seen with acausal algorithms is due to “smearing” of post-stimulus data: evidence from simulations

Furthermore, if the ERPs for detected and undetected stimuli differ, as is the case in our data, their distorting effects on the phase estimates at stimulus onset may also differ (Sauseng et al., 2007). Thus, it is possible that the apparent dependence of detection probability on δ-phase seen with acausal algorithms (Figure 5B) is simply the result of phase distortion at stimulus onset by “smearing” of post-stimulus data. We examined this possibility by simulations.

For this purpose, we generated 20,000 sets of 2 s long segments of sine waves of all integer frequencies within the δ-band (i.e., 1, 2, 3, and 4 Hz), each with an amplitude equal to the respective amplitude in our real data, as extracted from a 1.024-s pre-stimulus window by means of FFT (including Hanning window). Four sine waves, each with a different frequency, were then added, resulting in 20,000 summed waveforms. Since the phases of the sine waves were drawn from a uniform distribution, the distribution of the phases across the 20,000 realizations was also uniform, at any arbitrary time point. We defined the center of the segments as *t* = 0. We then added to each of the 20,000 summed waveforms the average ERP (cf. Figure 4A), either that for hits or that for misses, with equal probability, and as if tone onset had occurred at *t* = 0. From these simulated data, we extracted phase at *t* = 0 by means of causal and acausal BP filtering and HTF, just as described above for the real data. In addition to the 2-s window, we also used a 1-s window centered at *t* = 0 in combination with the acausal filter to substantiate our findings. The “detection probabilities” as a function of δ-phase at “tone onset,” resulting from these analyses, are shown in Figure 7 (black lines), where they can be compared with those derived from the real data and by the same algorithms (gray lines). Only with the causal filter, “detection probability” is independent of δ-phase (Figure 7A), as it should be. With the acausal filter and both peri-stimulus time windows, “detection probabilities” are sinusoidal functions of δ-phase, with “preferred” and “worst” phases, very similar to those obtained from the real data (Figures 7B,C).

Our simulation is clearly an oversimplification. Simulating ongoing brain activity as a sum of a few sine waves of specific frequencies and amplitudes is somewhat simplistic. Also, in real data the ERP and ongoing oscillations might not be purely additive (see Makeig et al., 2002; Kruglikov and Schiff, 2003; Sauseng et al., 2007; Thorne et al., 2011). Still, our simulation provides “proof of concept” that an apparent dependence of detection probability on the phase of δ-oscillations can arise from contamination by post-stimulus signals. Thus, our simulation strongly suggests that the dependence of detection probability on δ-phase as seen with acausal algorithms (Figures 5B and 7B,C) is indeed an artifact resulting from “smearing” of the ERPs, which differ for hits and misses.

##### EEG α-phase and tone detection

**Figure 8:**
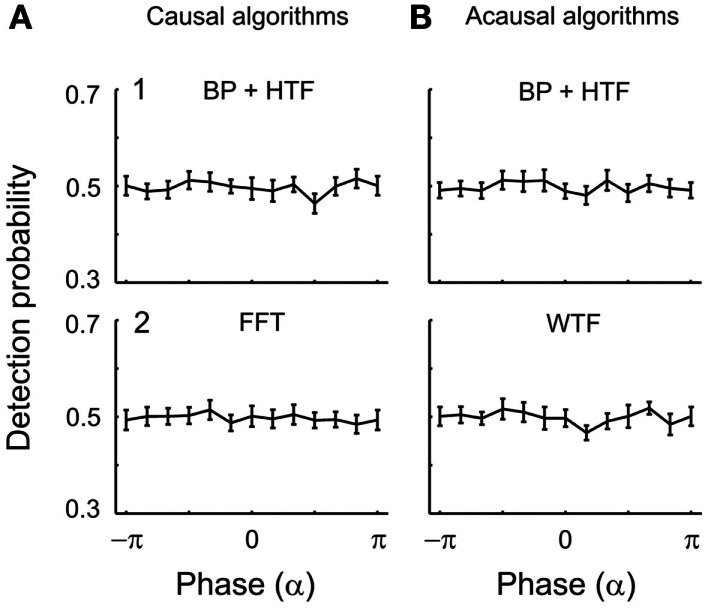
**Mean detection probability is independent of α-phase at the time of tone onset**. In **(A)**, causal algorithms, and in **(B)**, acausal algorithms were selected to estimate phase. The algorithms were (1) BP filtering (7–12 Hz) followed by HTF and (2) FFT or WTF (both shown for the representative frequency of 7 Hz). Other conventions as in Figure 5.

**Figure 9:**
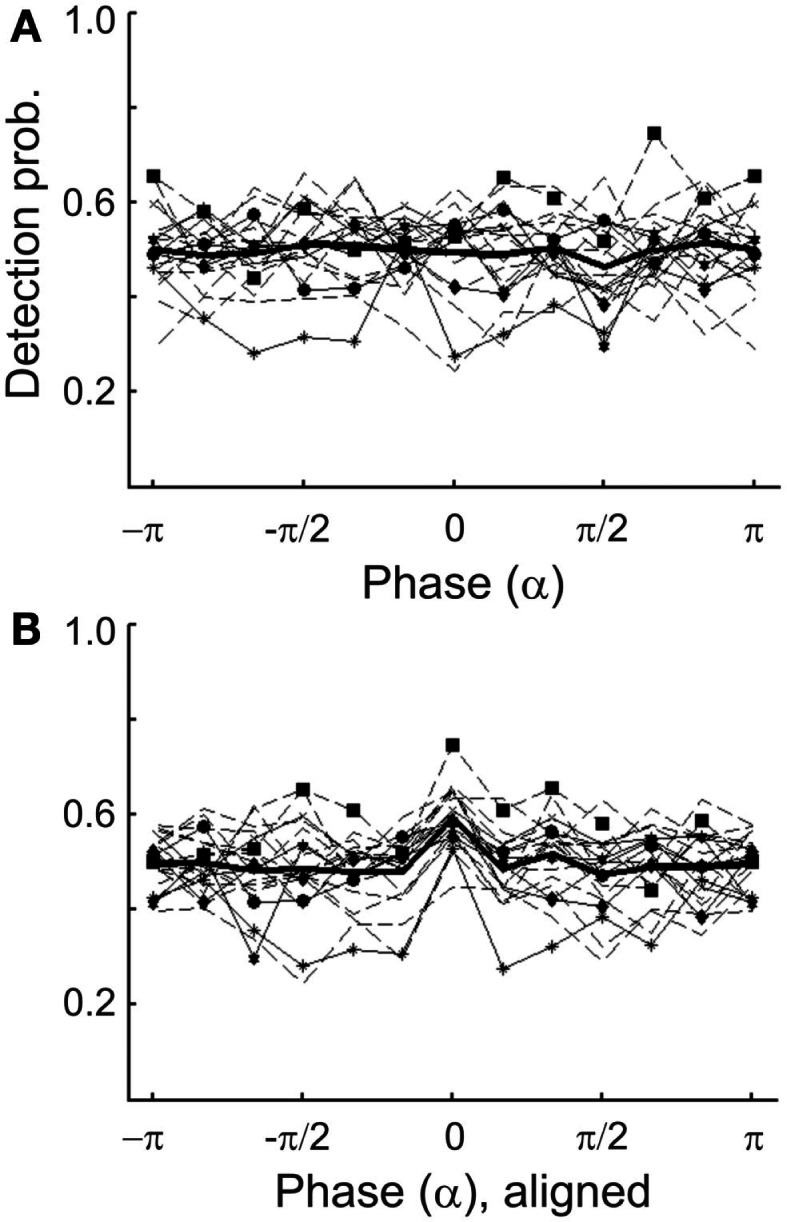
**(A)** Detection probability of individual subjects as a function of the estimated α-phase at tone onset (thin lines) in Experiment 1. Phase was estimated by causal BP filtering (7–12 Hz) followed by HTF. The mean function (thick line) is the same as that in Figure 8A1. The distribution of detection probability of six subjects deviated significantly (*p* < 0.05) from uniformity (different symbols; data from other subjects: broken lines). **(B)** Same individual functions as in **(A)**, but aligned such that the maximum detection probability of each subject coincides with the center bin.

Analogous analyses were performed with respect to the phase of oscillations in the α-band and the results are shown in Figures 8 and 9. Figure 8A shows the outcomes when α-phases at tone onset were estimated using causal algorithms. Here, the distributions of the mean detection probability across phase do not differ from uniformity [BP/HTF: *z*(415) = 0.11, *p* = 0.90; FFT: *z*(415) = 0.07, *p* = 0.93]. For each phase bin, detection probability is about 50%.

Similarly to what was observed for δ-phase, we found significant departures from uniformity of the distributions of detection probability across α-phase when examined at the individual level, in 6 of the 20 subjects (*p* < 0.05; Figure 9A). Again, after aligning the individual functions such that their maxima fall into the same bin (centered at 0), the mean detection probability, of course, reveals a peak in that bin (Figure 9B). However, as for δ-phase, there is no “worst” phase opposite the “preferred” phase. Instead, apart from the peak the function is rather flat. Also, Rayleigh’s Test provides no reason to reject the null hypothesis of a uniform phase distribution [*z*(415) = 0.57, *p* = 0.57].

Unlike what was observed for δ-phase, mean detection probability was independent of α-phase, also when derived with acausal algorithms (Figure 8B). Rayleigh’s Test yielded no reason to reject the null hypothesis [zero-phase BP/HTF: *z*(415) = 0.06, *p* = 0.94; WTF: *z*(415) = 0.17, *p* = 0.84].

We also performed simulations, analogously to those described above for the δ-band. Irrespective of the analysis window, and irrespective of whether a causal or acausal BP filter was applied to the simulated data, the “detection probability” was independent of α-phase (not shown), just as seen in the real data. Obviously, “smearing” of the ERP does not create an apparent dependence of detection probability on α-phase, presumably because the dominant stimulus-locked frequency in the ERP falls within the δ-band and not the α-band (Basar-Eroglu et al., 1992; Polich, 2007; Ergen et al., 2008; Doege et al., 2010). This notion is supported by the absence of “smearing,” when single trials are subjected to zero-phase filtering in the α-band before averaging them in order to reveal the ERP (Figure 4D, light gray).

##### Other frequency bands and tone detection

We repeated the analyses described above for several other frequency bands (θ, lower β, upper β, and lower γ; See Materials and Methods), but found no influence of phase on detection probability in any of these bands. Taken together, our results strongly suggest that under our experimental conditions tone detection is independent of EEG phase.

#### Effect of power

In the visual system, the effects of α-phase on stimulus detection are most pronounced when the power in that frequency band is high (Mathewson et al., 2009; Jensen et al., 2012). Presumably, high α-power is a prerequisite for restricting the time windows for neuronal firing and so for generating rhythmic neural activity (Klimesch et al., 2007). In the auditory system, it has been reported that target detection depends on the power of δ/θ-band oscillations at and before target onset, with detection rates being higher when power is low (Ng et al., 2012). It is thus conceivable that in our data a dependence of tone detection on EEG phase is only disguised by inclusion of many low- (or high-) power trials and that it might emerge when the analyses are restricted to high- (or low-) power trials. We therefore determined the power in each frequency band of every trial in the 1.024 s pre-stimulus time interval ending at tone onset (See Materials and Methods). We then divided the trials, for each frequency band separately, into high- and low-power ones, using a median split. For each group of trials, detection probability was calculated as a function of phase. The distributions of detection probability across phase derived from high- and low-power trials were not significantly different (for all bands *p* > 0.10; Kuiper’s Test). Recently, it has been reported that θ- (Lakatos et al., 2005; Schroeder et al., 2008), α- (Gomez-Ramirez et al., 2011), β- (Siegel et al., 2009; Saleh et al., 2010; Fiebelkorn et al., 2013), or γ-power (Bosman et al., 2009; Händel and Haarmeier, 2009) can be modulated by δ-phase. In that case, the 1.024-s pre-stimulus window would be too long for the extraction of power, as it includes several δ-cycles and therefore sections of the respective frequency bands that are characterized by both high and low power. Therefore, we repeated the analyses using shorter pre-stimulus time windows (e.g., 0.256 s) for the extraction of power. However, the outcome remained unchanged.

#### Phase and reaction time

Finally, we examined whether the EEG phase at the time of tone onset might have an effect on reaction time. For this purpose, hit trials of all subjects were pooled and divided into two groups, using a median split of reaction time. For each trial, phase at tone onset was derived by causal BP and HTF. For each group of hit trials (fast and slow reaction time), the circular mean phase was then calculated. Notably, mean phases during fast and slow reaction time trials did not differ (for all bands *p* > 0.20).

### Experiment 2

#### Rationale

In Experiment 1, the probability of detecting near-threshold tones was independent of the EEG phase, irrespective of power. Since in this experiment stimulus times were unpredictable for the subjects, it can be argued, following proposals of Schroeder et al. (2008, 2010), that the auditory system might have been operating in a “continuous mode” where “low-frequency oscillations are suppressed and the system is pushed as much as possible into a continuous state of high-excitability” (Schroeder and Lakatos, 2009). Also, in a recent study, it was reported that detection of visual targets depended on δ-phase when stimuli were presented at regular, but not at irregular, intervals (Cravo et al., 2013). We therefore conducted a second experiment to examine whether a dependence on phase emerges when the stimulus times are predictable so that the brain might be operating in a “rhythmic mode.” According to Schroeder and Lakatos (2009), operation in this mode “entails: (i) sensory cortical entrainment (phase-locking) to the temporal structure of an attended stream, (ii) alignment of “high-excitability” oscillation phases with events in the attended stream, and (iii) systematic enhancement of responses to attended events and suppression of events that occur out of phase with the attended events.”

In Experiment 2, we employed a “regular condition” where stimuli were presented at a constant ISI of 2 s and an “irregular condition” where stimuli were presented at irregular ISIs (drawn from the distribution of ISIs in Experiment 1; See Materials and Methods). From the irregular condition, we expected a corroboration of our findings in Experiment 1. In the regular condition, we expected an entrainment of EEG oscillations to the tones, i.e., the alignment of a “high-excitability” phase of oscillations corresponding to the frequency of stimulus presentation, i.e., 0.5 Hz, with tone onset. Phase entrainment would indicate an operation of the system in the “rhythmic mode.” Moreover, in this mode, we expected tone detection to depend on the phase of oscillations in common frequency bands, in particular at upper harmonics of 0.5 Hz (as phase entrainment has also been observed at upper harmonics of the frequency of stimulus presentation; cf. Gomez-Ramirez et al., 2011; Power et al., 2012).

#### Behavioral results

The mean of the thresholds for the tone stimulus across the 21 subjects was 9.1 dB SPL. Despite similar individual mean detection probabilities in the regular (53.3 ± 6.8%) and irregular conditions (49.8 ± 5.5%), due to the fine sound level adjustments (see Materials and Methods), the individual median reaction times were significantly shorter [Wilcoxon signed rank test, *z*(21) = 3.57, *p* < 0.01] in the regular condition. In this condition, the median of pooled reaction times was 0.42 s (interquartile range 0.35–0.52 s) and in the irregular condition 0.47 s (interquartile range 0.39–0.57 s).

#### Phase entrainment in the regular condition

**Figure 10:**
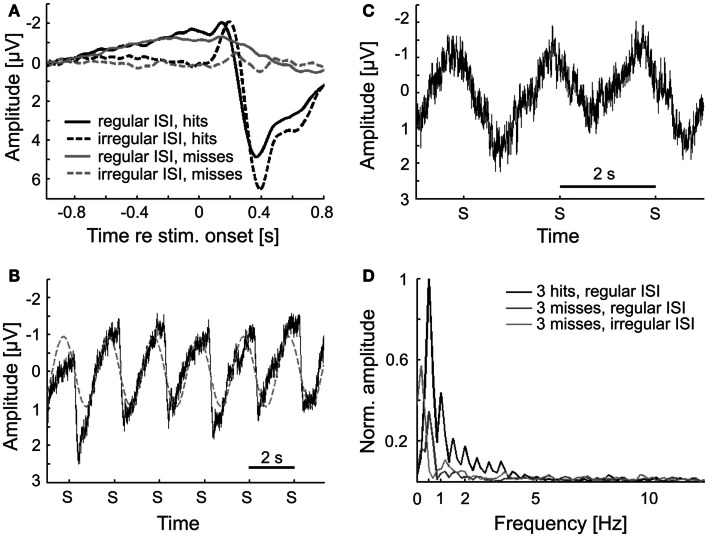
**Regular but not irregular stimulus presentation results in a phase entrainment to the stimulus presentation**. **(A)** ERPs for hits and misses. The ERP is virtually absent for misses, whereas pronounced early negative and late positive components are visible for hits. A slow negative shift is present in the regular condition, for both hits and misses. **(B)** The EEG signal, averaged within and across subjects and across hits and misses, to six subsequent stimuli (stimulus onset marked as “S”) reveals an apparent periodic “reset” of the slow negative shift by the ERP every 2 s (gray dashed line shows a fit to the data with a sine function of 0.5 Hz). **(C)** The average EEG signal to three successive misses reveals an oscillation at the frequency of stimulus presentation. As the ERP is essentially absent for misses (cf. **A)**, the observed signal suggests phase entrainment, evoked by the rhythmic stimulation, rather than a regular repeat of negative shift and ERP. **(D)** Amplitude spectra of the average EEG signals around intervals comprising three consecutive hits or three consecutive misses, both in the regular condition, and three consecutive misses in the irregular condition of Experiment 2. All spectra are normalized with respect to the peak, at 0.5 Hz, of the first one.

Figure 10A shows the ERP and pre-stimulus activity for hits and misses, separately for the regular and the irregular condition. For clarity, we refrain from showing the ERPs separately for the three sound levels, but note that the results were similar to those in Experiment 1 in that both early negative (at 200 ms) and late positive (at 400 ms) components increased in amplitude with increasing sound level, in both conditions. For misses, the ERP was virtually absent, in both conditions. For hits, irregularly occurring stimuli evoke larger negative and positive components than regularly occurring stimuli, a finding that can be attributed to the lower level of expectancy in the irregular condition (Duncan et al., 2009). For both hits and misses, a slow negative shift of the pre-stimulus activity can be observed, but only in the regular condition.

Figure 10B shows the EEG signal recorded to six successive stimuli from the regular condition (i.e., over 12 s), averaged within and across subjects, and irrespective of whether the stimuli were detected or not. It appears that the slow negative shift is “reset” by each ERP. Of course, since the stimuli were presented at a fixed interval of 2 s, the dominant frequency in the 12-s segment is 0.5 Hz (illustrated by the fit of a 0.5-Hz oscillation to the data in Figure 10B). Hence, it remains unclear whether the signal in Figure 10B reflects phase entrainment at the frequency of stimulus presentation or merely a continuous “cycle” between negative shift and ERP (see also Lakatos et al., 2013, for a dissociation of entrainment and ERP). Figure 10C tries to address this issue and to disentangle regular ERPs and possible entrainment. The trace shows the EEG signal (averaged within and across subjects) recorded around the presentation of three successive stimuli that, however, were all missed by the subjects. We cannot exclude the possibility that, although the stimuli were missed, some of them may have triggered ERPs, but in the average trace ERPs cannot be discerned, consistent with the results in Figure 10A. Still, the EEG signal clearly oscillates with a frequency of 0.5 Hz. This is illustrated by the prominent peak at 0.5 Hz in the normalized amplitude spectrum of these data, shown in Figure 10D (dark gray line), although, as expected, the peak is less pronounced than that in the spectrum extracted from EEG signals comprising three consecutive hits (black line). The latter also exhibits pronounced peaks at frequencies corresponding to the upper harmonics of 0.5 Hz, obviously caused by the ERPs. The stimulus onsets (marked as “S” in Figures 10B,C) coincide with a very similar phase of the 0.5 Hz oscillation (just past the negative maxima of the EEG signal). Therefore, the EEG signal can be said to be entrained to the stimulus presentation rate, as expected from a “rhythmic mode.” Our findings suggest that, even at threshold sound levels where about half of the stimuli are missed, the neural system adjusts to the rhythmic stimulation (here every 2 s), manifest as phase entrainment. In contrast, when stimuli are presented in an unpredictable fashion, supposedly evoking operation in a “continuous mode” (Schroeder and Lakatos, 2009), low-frequency oscillations are less pronounced. This is reflected in the average amplitude spectrum of the EEG signals comprising three consecutive misses in the irregular condition, also shown in Figure 10D (light gray line). This spectrum contains no peak at 0.5 Hz.

#### Phase entrainment: relation to tone detection and reaction time

Of course, we expect the entrainment seen in the regular condition during brief periods of successive misses to be present also during hits. However, this cannot be shown directly due to contamination by the regular ERPs. The phase of the dominant 0.5-Hz component at tone onset cannot be reliably extracted from a 1-s pre-stimulus window (the maximal window length that allowed estimating phases without including the ERP in the analysis), since a 1-s window covers only half the period of a 0.5-Hz oscillation. Given the entrainment, our data do not allow examining whether the phase of the 0.5-Hz oscillation at stimulus onset correlates with detection probability. There is no way out of this dilemma. Nevertheless, phase entrainment may have a beneficial influence on reaction time (e.g., Lakatos et al., 2008; Schroeder and Lakatos, 2009; Stefanics et al., 2010; Thut et al., 2011; Cravo et al., 2013), because reaction times were significantly shorter in the regular than in the irregular condition of Experiment 2, although sound levels were adjusted such that detection probability was about 50% in both conditions (cf. Behavioral results).

#### Effect of power

We also asked whether the power of the 0.5-Hz oscillation might be correlated with detection probability and reaction time. As a surrogate measure of that power we quantified the steepness of the negative shift prior to tone onset. The steeper the shift, the larger is the amplitude, and the higher the power. The steepness of the negative shift was quantified by subtracting the mean amplitude of the signal during the first half of the 400-ms pre-stimulus period from the mean amplitude during the second half. The steeper the shift, the more negative is this difference, *D*. After sorting the single trials according to *D*, we divided the data into 20 bins and calculated the mean detection probability and median reaction time for each bin. There was no significant correlation between *D* and detection probability (*r* = −0.08, *p* = 0.12) or *D* and reaction time (*r* = 0.06, *p* = 0.25). Nevertheless, *D* was more negative for hit trials (−0.44 ± 0.66 μV) than for miss trials (−0.09 ± 0.58 μV). Although this difference was not significant (*z* = 1.83, *p* = 0.07, Wilcoxon ranksum test), it could point to a relationship between the power of the entrained 0.5-Hz oscillation and stimulus detection.

#### Entrainment in the δ-band and tone detection

**Figure 11:**
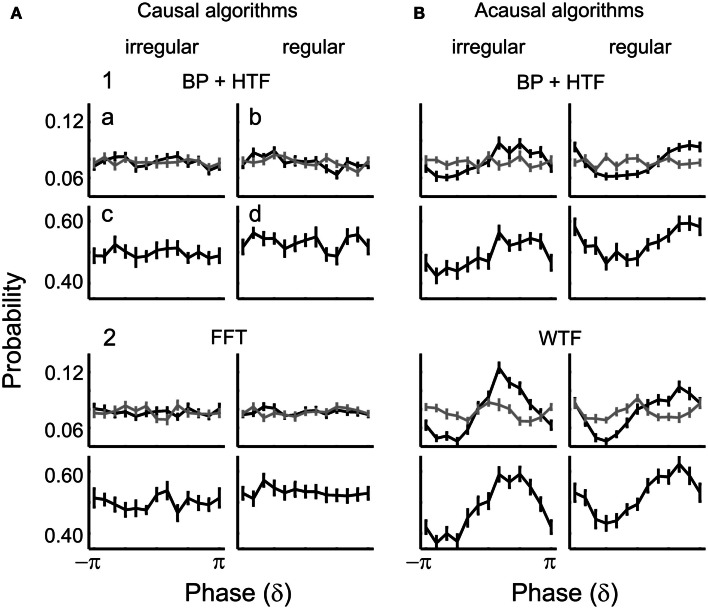
**Distributions of δ-phase at tone onset (a,b) for hits (black) and misses (gray), and of corresponding detection probabilities (c,d) in the irregular (a,c) and regular conditions (b,d) of Experiment 2**. Phases extracted by causal and acausal algorithms are shown in **(A,B)**, respectively. Rows 1–2 show the results obtained by (1) BP (1–4 Hz) and HTF, and (2) FFT or WTF (both for a frequency of 2 Hz).

We also asked whether the phase of higher frequencies, including harmonics of the 0.5-Hz oscillation, might entrain to the stimuli in the regular condition and correlate with detection probability. This was not the case. Figure 11 shows the distributions of δ-phase at tone onset (a,b) and the corresponding detection probabilities (c,d) for the irregular (a,c) and regular (b,d) condition, for both causal (Figure 11A) and acausal (Figure 11B) algorithms.

For causal algorithms, there is no sign of entrainment, neither in the irregular nor the regular condition (Figures 11A1,2a,b) (*p* ≥ 0.5 for both hits and misses in both conditions). Also, there was no dependence of detection probability on phase, neither in the irregular (Figures 11A1,2c) nor in the regular condition (Figures 11A1,2d) (*p* > 0.6 for both algorithms and conditions). Thus, Experiment 2 corroborates our finding in Experiment 1 that detection probability is independent of δ-phase when stimuli are presented at irregular intervals. In addition, Experiment 2 reveals that detection probability is independent of δ-phase, even when stimuli are presented at regular intervals. The same was true with respect to the other frequency bands (θ, α, lower α, upper α, lower β, upper β, and lower γ). Also as in Experiment 1, there was no effect of power: the results did not differ between high- and low-power trials, in any frequency band.

For acausal algorithms, there appeared to be entrainment as well as a dependence of detection probability on phase, with a preference for the phase aligned with tone onset (Figure 11B). However, these effects are again caused by “smearing” of the ERP. This “smearing” leads to a non-uniform phase distribution at stimulus onset for hits only (Figures 11B1,2a,b; black lines) (most *p* < 0.01), but not for misses (gray lines) (all *p* > 0.6), because there is no ERP that can be “smeared” (Figure 10A; see also Sauseng et al., 2007). Consequently, since the phase distribution is biased toward a certain phase for hits only, the detection probability appears highest at that “biased” phase, resulting in non-uniform distributions of detection probability across phase (Figures 11B1,2c,d) (all *p* ≤ 0.01).

## Discussion

The phase of EEG oscillations has been reported to modulate the probability of detecting near-threshold stimuli. In the visual system, the phase of α-oscillations, thought to reflect periodic “pulsed inhibition” in the neural network (Klimesch et al., 2007; Mathewson et al., 2009; Mazaheri and Jensen, 2010; Jensen et al., 2012), seems critical (Busch et al., 2009; Mathewson et al., 2009; Busch and VanRullen, 2010; Dugué et al., 2011). In the auditory system, lower frequencies seem also important. Detection of near-threshold auditory targets in noise or of gaps in ongoing sounds has been reported to depend on the phase of EEG δ-, θ-, or α-oscillations presumed to be driven by the background sounds (Henry and Obleser, 2012; Ng et al., 2012) or by oscillating transcranial direct current stimulation (o-tDCS; Neuling et al., 2012). In our study, we examined whether the probability of detecting near-threshold sounds in quiet depends on the phase of ongoing EEG oscillations. Based on the above reports, we had expected to find effects of phase on detection. However, in our hands, detection was independent of phase in common frequency bands, irrespective of whether stimuli were presented at irregular or regular intervals. This negative result held true not only for electrode Cz, for which data are shown, but also for other electrodes.

### Stimulus design

Our finding is even more surprising given that our stimulus design seems optimal to reveal a dependence of detection probability on the phase of some EEG oscillation, if it existed. First, we presented the stimuli at near-threshold sound levels, yielding an average detection probability of about 50%. Here, the psychometric function is steepest. Consequently, a given subtle change in the input (e.g., in the sound level or – if relevant – in the phase at stimulus onset) would have the largest effect on detection probability. Second, we used tones whose brief durations (12.48 ms) correspond to only small fractions of the periods of EEG oscillations. If detection were some sinusoidal function of the phase of such oscillations, then the largest difference in detection would result when brief stimuli are presented at the opposing “preferred” and “worst” phases. The longer the stimulus, the larger is the stimulus fraction that would coincide with more neutral phases, thus reducing the effect of phase on detection probability. Thus, if there were some significant effect of phase on the detectability of near-threshold sounds, we should have detected it.

### Continuous versus rhythmic mode and entrainment

In our first experiment, stimulus timing was unpredictable. The auditory system may therefore have been operating in the “continuous mode” of constant neuronal excitability and sensitivity (Schroeder et al., 2008, 2010; Schroeder and Lakatos, 2009). If so, our finding that detection is independent of phase, irrespective of whether power is relatively high or low, is actually expected from the definition of this mode. In line with this notion, the amplitude of the 0.5-Hz oscillation in the irregular condition was lower than in the regular condition (where stimuli were presented every 2 s) (Figure 10D). In the regular condition of our second experiment, stimulus timing was regular and predictable. Here, the system could have adapted to the regular stimulation and operated in the “rhythmic mode,” characterized by alternating states of low and high neuronal excitability and reflected in the phase of EEG oscillations (Schroeder et al., 2008, 2010; Schroeder and Lakatos, 2009). As an indicator for operation in the “rhythmic mode,” entrainment of δ-oscillations in the auditory system to auditory or visual stimuli, repeated periodically, or almost periodically, has been reported (Lakatos et al., 2005, 2008; Schroeder et al., 2008; Besle et al., 2011; Gomez-Ramirez et al., 2011). Phase entrainment has been suggested to function as a mechanism of attentional selection (Lakatos et al., 2008; Schroeder and Lakatos, 2009; Schroeder et al., 2010), thereby also reducing reaction times (Stefanics et al., 2010; Cravo et al., 2013), or facilitating speech comprehension (Ghitza, 2011; Peelle and Davis, 2012). Indeed, we observed an EEG signal which oscillated with a period identical to the constant ISI (2 s; Figure 10B). To clarify whether this oscillation reflected true entrainment or was solely due to regularly triggered ERPs (Lakatos et al., 2013), we examined periods of successive misses and found the oscillation to be present also during these periods (Figure 10C). Of course, we cannot completely rule out that the tones, even when undetected, evoked a weak response that contributed to this oscillation. Since the short stimuli all coincided with a very similar phase of the 0.5 Hz oscillation, the EEG signal in the regular condition can be considered to be entrained to the stimulus rate, as expected from a “rhythmic mode.” Thus, entrainment can occur at threshold stimulus levels where only about half of the stimuli are detected. Previous studies reporting phase entrainment used targets or driving background stimuli far above threshold (Lakatos et al., 2005, 2008; Schroeder et al., 2008; Saleh et al., 2010; Stefanics et al., 2010; Besle et al., 2011; Gomez-Ramirez et al., 2011; Henry and Obleser, 2012; Cravo et al., 2013).

The amplitude of the entrained oscillation prior to stimulus onset was higher in hit trials than in miss trials. Although this difference was not statistically significant, it could nevertheless be viewed as weak evidence for a relationship between stimulus detection and power of an entrained oscillation, when the system operates in the “rhythmic mode,” in line with theoretical considerations (Lakatos et al., 2008; Schroeder and Lakatos, 2009; Schroeder et al., 2010). However, and in contrast to our expectations, we found no effect of EEG phase on detection probability for any of the frequencies for which phase at tone onset could be derived (δ and above). This could indicate that the phase of commonly analyzed frequencies does not have an impact on tone detection, not only during the irregular condition, i.e., in the presumed “continuous mode,” but also in the regular condition, i.e., in the presumed “rhythmic mode.” Of course, we cannot exclude the possibility that phase does matter, but that we are simply not able to detect its influence (see next section).

Operation in the “rhythmic mode” might be beneficial for stimulus anticipation and for executing requested motor responses, since reaction times were significantly shorter in the regular than in the irregular condition. Therefore, one may argue that the phase entrainment of the 0.5-Hz oscillation may have a beneficial effect on reaction times (see, e.g., Stefanics et al., 2010; Bonnefond and Jensen, 2012; Cravo et al., 2013). However, correlation does not mean causality. The shorter reaction times in the regular condition may have resulted from the superior predictability of stimulus times or the shorter mean ISI in this condition. Both regular and irregular conditions can be viewed such that each detected stimulus acts as a “warning signal” announcing the next signal requiring a response. Reaction times depend on factors such as the duration of the time between warning signal and response signal and whether that duration is fixed or varies from trial to trial (Luce, 1986). Nevertheless, it is possible that the entrainment of the EEG signal in the regular condition constitutes a physiological correlate of the superior predictability or even reflects the prediction mechanism (e.g., some clock; Saleh et al., 2010; Cohen, 2011; Cravo et al., 2011). In the pre-stimulus activity of the regular condition, the 0.5-Hz oscillation is reflected as a slow negative shift (see Figure 10A). Similar slow negative shifts before the occurrence of expected events have been observed repeatedly and discussed as a reflection of stimulus anticipation or motor preparation (Walter et al., 1964; Brunia and van Boxtel, 2001; see also Brosch et al., 2011).

### Comparison of visual and auditory systems

In contrast to our results, Busch et al. (2009) reported a dependence of the detection probability of a near-threshold visual stimulus on the EEG phase in the lower α-band, even though the cue-target interval was variable. It is therefore conceivable that α-oscillations play a prominent role in the visual system but not in the auditory system. This is supported by work of VanRullen and colleagues. VanRullen and MacDonald (2012) had subjects view flicker sequences. By reversely correlating the EEG signal with the luminance sequence, they found an oscillatory “echo” at 10 Hz lasting >1 s, but attempts to replicate these findings in the auditory domain failed (Ilhan and VanRullen, 2012). Only one recent study reported that thresholds for detecting a brief auditory signal in noise depended on the phase of α-oscillations entrained by o-tDCS (Neuling et al., 2012). However, it cannot be excluded that the small effects (±0.3 dB) result from periodic activation of middle ear muscles by the transcranial current stimulation. Taken together, these findings suggest that α-oscillations, a reflection of an active inhibitory control mechanism in the visual system (Klimesch et al., 2007), either do not play a major role in the auditory system or that their role for audition is not yet revealed. It should also be kept in mind here that volume conduction through the skull often presents a problem for the interpretation of EEG recordings (Stinstra and Peters, 1998; Haufe et al., 2013). The signature of neural activity in auditory cortex might be more attenuated, or contaminated by volume conduction, in scalp EEG recordings than that in somatosensory or visual cortex, due to the “nested” position of the auditory cortex in the lateral sulcus. Such attenuation and contamination might hinder or even prevent the detectability of possible phase effects. Intracranial recordings might be necessary, and would be interesting, to resolve this issue.

Two other recent studies (Henry and Obleser, 2012; Ng et al., 2012) reported that the phase of oscillations, in the δ/θ-range and entrained by background sounds, have significant effects on the detection of auditory targets (brief sounds or gaps) in background sounds. Ng et al. (2012) reported the phase dependence to be stronger for hits than for misses, an intriguing asymmetry which, however, may be explained by biased sampling (see VanRullen and McLelland, 2013). It is also noteworthy that Ng et al. used acausal filters (B. S. W. Ng and C. Kayser, personal communication). Henry and Obleser (2012) used a wavelet convolution – an acausal algorithm as well – to estimate phase.

### Methodological considerations

When we used acausal algorithms (zero-phase BP filtering and HTF or WTF) to estimate the phase at stimulus onset, an apparent dependence of detection probability on the phase of δ-oscillations at that time emerged (Figures 5B, 7B,C, and 11B). The dependence appears sinusoidal with detection probability varying between 40 and 60%, similar to what has been reported for visual and somatosensory stimuli (Monto et al., 2008; Busch et al., 2009; Mathewson et al., 2009; Busch and VanRullen, 2010; Cravo et al., 2013) as well as for auditory targets in background sounds (Henry and Obleser, 2012; Neuling et al., 2012; Ng et al., 2012). Also, we found an apparent phase entrainment in the δ-band (Figures 11B1,2a,b), similar to results of other studies (Will and Berg, 2007; Saleh et al., 2010; Stefanics et al., 2010; Gomez-Ramirez et al., 2011; Henry and Obleser, 2012; Power et al., 2012; Cravo et al., 2013) along with seemingly superior detection at the “entrained” phase (Figure 11B). Both acausal time windows used by us to estimate phase, however, include the ERP. Consequently, acausal BP filtering or WTF will “smear” the ERP back in time and affect the estimate of the phase at stimulus onset (Figure 4D; dark gray). In our data, the ERPs for hits and misses differed considerably with respect to shape, amplitude, and peak latencies (Figures 4 and 10), and these differences may generate an apparent dependence of detection probability on phase, even if in reality there is no dependence at all. In fact, our analyses using a causal time window and our simulations (Figure 7) suggest this to be the case. The apparent dependence on δ-phase is an artifact resulting from contamination by post-stimulus differences in the ERPs for hits and misses.

It may be argued that the “principal frequency” of the ERP is higher when stimuli are presented at supra-threshold levels (Musiek et al., 2005; Garinis and Cone-Wesson, 2007), so that phase distortion might not affect the δ-range. Also, in a passive listening condition, the P300 might be smaller than in an active listening condition (Polich, 2007), so that in data from the former condition the “oscillatory” component of the ERP might not affect phase estimation at tone onset by acausal algorithms as strongly as in our data. We tried to investigate these possibilities by running simulations similar to those described in Section “Apparent Dependence of Detection on δ-Phase Seen with Acausal Algorithms is Due to “Smearing” of Post-stimulus Data: Evidence from Simulations”. For the present purpose, we added sine waves with all integer frequencies between 1 and 10 Hz and amplitudes equal to the respective average amplitudes extracted from 1.024-s pre-stimulus windows of our real data in Experiment 1. We then added a simulated ERP (plus noise), with the N1-P2 component having a frequency of 6 Hz (i.e., in the θ-range) and an amplitude of ±4 μV (i.e., similar to the amplitudes shown in Figure 4A). Acausal BP filtering between 4 and 8 Hz was then applied before phase extraction by HTF. Notably, however, phase estimation at stimulus onset was still affected by “post-stimulus” data. Furthermore, we found phase distortion not only in the θ-band, but also in the δ-band, indicating that even if the “principal frequency” of the ERP falls into the θ-range, estimation of δ-phase by acausal algorithms can still be affected by post-stimulus data. This was not the case when phase was extracted by causal algorithms. We conclude that acausal algorithms should be used with great caution and that – at least in paradigms similar to those in our study – potential artifacts can only be reliably excluded when causal algorithms are used to extract phase at stimulus onset.

### Conclusion

We found no evidence for a dependence of the detection of sounds presented at near-threshold levels on the phase of EEG oscillations in frequency bands commonly described in the literature, irrespective of whether the sounds occurred at unpredictable or predictable times. Only when we analyzed the data using acausal, though common, algorithms, both phase entrainment and an apparent dependence of detection probability on the δ-phase emerged, similar to those described in previous publications. In our study, the dependence is an artifact from the ERP which differs for hits and misses. We do not suggest that similar artifacts would have necessarily produced the phase dependences in previous studies, particularly since some have gone at lengths to rule them out (e.g., Busch et al., 2009; Stefanics et al., 2010; Fiebelkorn et al., 2013). Our analyses are intended to raise awareness of this common problem which, if ignored, might bias and mislead this exciting field of research.

## Conflict of Interest Statement

The authors declare that the research was conducted in the absence of any commercial or financial relationships that could be construed as a potential conflict of interest.
